# Evaluation of Feature Selection Methods for Mammographic Breast Cancer Diagnosis in a Unified Framework

**DOI:** 10.1155/2021/6079163

**Published:** 2021-10-04

**Authors:** Chun-jiang Tian, Jian Lv, Xiang-feng Xu

**Affiliations:** ^1^Department of Radiology, Tianjin Hospital of ITCWM Nankai Hospital, Tianjin 300100, China; ^2^Department of Radiology, Tianjin Central Hospital of Obstetrics and Gynecology, Tianjin 300100, China

## Abstract

Over recent years, feature selection (FS) has gained more attention in intelligent diagnosis. This study is aimed at evaluating FS methods in a unified framework for mammographic breast cancer diagnosis. After FS methods generated rank lists according to feature importance, the framework added features incrementally as the input of random forest which performed as the classifier for breast lesion classification. In this study, 10 FS methods were evaluated and the digital database for screening mammography (1104 benign and 980 malignant lesions) was analyzed. The classification performance was quantified with the area under the curve (AUC), and accuracy, sensitivity, and specificity were also considered. Experimental results suggested that both infinite latent FS method (AUC, 0.866 ± 0.028) and RELIEFF (AUC, 0.855 ± 0.020) achieved good prediction (AUC ≥ 0.85) when 6 features were used, followed by correlation-based FS method (AUC, 0.867 ± 0.023) using 7 features and WILCOXON (AUC, 0.887 ± 0.019) using 8 features. The reliability of the diagnosis models was also verified, indicating that correlation-based FS method was generally superior over other methods. Identification of discriminative features among high-throughput ones remains an unavoidable challenge in intelligent diagnosis, and extra efforts should be made toward accurate and efficient feature selection.

## 1. Background

Feature selection (FS) or variable selection plays an important role in intelligent diagnosis. It is used to identify a subset of features or to weight the relative importance of features in target representation that makes a computer-aided diagnosis model cost-effective, easy to interpret, and generalizable. So far, FS methods have been explored in target recognition [1], logistic regression [2], disease detection and diagnosis [3–6], bioinformatics [7–9], and many industrial applications [10–12].

According to the interaction with machine learning classifiers (MLCs), FS methods can be broadly categorized into three groups [13–16]: (1) filter method that selects features regardless of MLCs. It estimates the correlation between quantitative features and target labels, and the features with strong correlations to data labels are further considered. This kind of approach is efficient and robust to overfitting; however, redundant features might be selected. (2) Wrapper method that uses learning algorithms to select one among the generated subsets of features. It allows for possible interactions between features, while it considerably increases computation time, in particular with a large number of features. (3) Embedded method that is similar to the wrapper method, while it performs FS and target classification simultaneously.

Few studies have addressed the efficiency comparison of FS methods. Wang et al. [17] have compared six filter methods, such as *chi*-square [18] and RELIEFF [19], and ranked features were further analyzed by using different MLCs and performance metrics. Experimental results indicated that the selection of performance metrics is crucial for model building. Furthermore, Ma et al. [20] have examined eight FS methods and found that support vector machine- (SVM-) based recursive feature elimination [6] is a suitable approach for feature ranking. In addition, they strongly suggested performing FS before object classification. Moreover, Cehovin and Bosnic [21] have evaluated five methods and discovered that RELIEFF [19] in combination to random forest (RF) [21] achieves highest accuracy and reduces the number of unnecessary attributes. Vakharia et al. [12] have compared five FS methods for fault diagnosis of ball bearing in rotating machinery, reporting that both the combination of Fisher score and SVM [22] and the combination of RELIEFF and artificial neural network (ANN) [23] have good accuracy. Additionally, Upadhyay et al. [24] have explored three methods to select informative features in wavelet domains. Specifically, they used the least square SVM and discovered that Fisher score has the highest discrimination ability for epilepsy detection.

This study performed an evaluation of FS methods, and a total of 8 filter methods, 1 wrapper method, and 1 embedded method were involved. Specifically, the evaluation was conducted in a proposed unified framework where features were ranked and incrementally added; RF was the classifier, and 4 metrics were used to assess the classification performance. Notably, the digital database for screening mammography (DDSM) [25] was investigated which contains 1104 benign and 980 malignant lesions. In the end, a test-retest study was concerned and the reliability of built models was discussed.

## 2. Methods

### 2.1. Data Collection

The DDSM is one of the largest databases for mammographic breast image analysis [25–27], which is available online (http://www.eng.usf.edu/cvprg/Mammography/Database.html). The database includes 12 volumes of normal cases, 16 volumes of benign cases, and 15 volumes of malignant mass lesion cases. Each case is represented by 6 to 10 files, i.e., an “ics” file, an overview 16-bit portable gray map (PGM) file, four image files compressed with lossless joint photographic experts group (LJPEG) encoding, and a zero to four overlay files.

Using the toolbox DDSM Utility (https://github.com/trane293/DDSMUtility) [28], a total of 2084 histologically verified breast lesions (1104 benign and 980 malignant lesions) and 4016 mammographic images were obtained. Full details on how to convert the dataset from an outdated image format (LJPEG) to a usable format (i.e., portable network graphic) and on how to extract these outlined regions of interest are described in the toolbox manual.

### 2.2. Lesion Representation

Previous studies have suggested computational and informative features for mammographic lesion representation [29, 30]. In this study, 18 features were used to characterize breast mass lesions among which 7 features (mean, median, standard deviation, maximum, minimum, kurtosis, and skewness) represent the statistical analysis of mass intensity, 8 features (area, perimeter, circularity, elongation, form, solidity, extent, and eccentricity) describe the lesion shape, and 3 features (contrast, correlation, and entropy) are derived from the texture analysis using the grey-level cooccurrence matrix (GLCM) [31]. Full information to these quantitative features can be referred to [32].

### 2.3. Feature Selection Methods

In total, 10 feature selection methods (8 filter methods, 1 wrapper method, and 1 embedded method) were evaluated. Specifically, there were 6 methods based on unsupervised learning and 4 methods based on supervised learning (Table 1).

Brief description of each method is as below

- Correlation-based feature selection (CFS) was used to quantify the relationship between feature vectors using Pearson's linear correlation coefficient [33]. It takes the minimal correlation coefficient of one feature vector to the other feature vectors as the score which represents the information redundancy. Finally, features were sorted according to the scores in ascending order
- Feature selection via eigenvector centrality (ECFS) [34] recasts the FS problem based on the affinity graph and the nodes in the graph present features. It estimates the importance of nodes through the indicator of eigenvector centrality (EC). And the purpose of EC is to quantify the importance of a feature with regard to the importance of its neighbors and these central nodes are ranked as candidate features
- Infinite latent feature selection (ILFS) [35] is a probabilistic latent FS approach that considers all the possible feature subsets. It further models feature “relevancy” through a generative process inspired by the probabilistic latent semantic analysis [36]. The mixing weights are derived to measure a graph of features, and a score of importance is provided by the weighted graph for each feature, which indicates the importance of the feature in relation to its neighboring features
- Laplacian score (LAPLACIAN) [37] evaluates the importance of a feature by its power of locality preserving. It constructs a nearest neighbor graph to model the local geometric structure, and it seeks the features that respect this graph structure
- Least absolute shrinkage and selection operator (LASSO) [38] performs feature selection and regularization simultaneously and thus, it can balance prediction accuracy and model interpretability. LASSO is L_1_-constrained linear least squares fits, and the importance of each feature is weighted
- Feature selection using local learning-based clustering (LLCFS) [39] estimates the feature importance during the process of local learning-based clustering (LLC) [40] in an iterative manner. It associates a weight to each feature, while the weight is incorporated into the regularization of the LLC method by considering the relevance of each feature for the clustering
- RELIEFF [19] estimates the weight of each feature according to how well its value can differentiate between itself and its neighboring features [41]. Thus, if the difference in feature values is observed in a neighboring instance pair with the same class, its weight decreases; while if there are different classes, its weight increases
- ROC is an independent evaluation criterion [42] which is used to assess the significance of every feature in the separation of two labeled groups. It stands for the area between the empirical receiver operating characteristic (ROC) curve and the random classifier slope. Higher area value indicates better separation capacity
- Unsupervised feature selection with ordinal locality (UFSOL) [43] is a clustering-based approach. It proposes a triplet-induced loss function that captures the underlying ordinal locality of data instances. UFSOL can preserve the relative neighborhood proximities and contribute to the distance-based clustering
- Wilcoxon rank-sum test (WILCOXON) or Mann-Whitney *U* test is a nonparametric test [44]. It requires no assumption of normal distribution of feature values. The test provides the most accurate significance estimates, especially with small sample sizes and/or when the data do not approximate a normal distribution

Among these methods, 4 methods consider statistical analysis on differentiating each other features or on label classification (CFS, RELIEFF, ROC, and WILCOXON); 3 methods build a graph to map the relationship between features, and weights of features are quantified by the specific measure spaces (ECFS, ILFS, and LAPLACIAN); 2 methods concern data clustering (LLCFS and UFSOL) for feature weighting; and 1 method merges feature selection into a regularization problem to balance prediction accuracy and model interpretability (LASSO). During the procedure, FS methods put a weight to each feature and thus, these features can be ranked according to their weights from the most to the least important.

### 2.4. Performance Metrics

In this study, four metrics, the area under the curve (AUC), accuracy (ACC), sensitivity (SEN), and specificity (SPE), were used to quantify the classification performance [45]. In particular, AUC presents the overall capacity of a model in lesion classification and it refers to the area under the ROC curve.

Based on histological verification, true positive (TP) is the number of positive cases that were correctly predicted as “positive,” false negative (FN) represents the positive cases that were misclassified as “negative,” true negative (TN) represents the true negative cases that were predicted correctly, while false positive (FP) is true negative cases that were predicted as “positive.” ACC, SEN, and SPE can be formulated using the formula (1), (2), and (3), respectively.

(1) $$\begin{array}{c} \text{ACC}=\frac{\text{TP}+\text{TN}}{\text{TP}+\text{FN}+\text{FP}+\text{TN}}, \end{array}$$

(2) $$\begin{array}{c} \text{SEN}=\frac{\text{TP}}{\text{TP}+\text{FN}}, \end{array}$$

(3) $$\begin{array}{c} \text{SPE}=\frac{\text{TN}}{\text{TN}+\text{FP}}. \end{array}$$

### 2.5. Experiment Design

Given 2084 lesion cases (1104 benign and 980 malignant lesions) of 4016 mammographic images, we took one image per lesion in the test study (a total of 1104 benign images and 980 malignant images) and the remaining images (1017 benign lesion images and 915 malignant lesion images) were used to retest the trained diagnostic models in the test study. Specifically, in the test study, 400 benign lesion images and 400 malignant lesion images were randomly picked for training and the other images were used for testing. The experiment was carried out 100 times, and performance metrics were reported on average.

RF is used as the classifier in this study. It is an ensemble learning method that has been widely applied for prediction, classification, and regression [20, 21, 46], and Strobl et al. utilized it to measure the variable importance [47]. The most important parameter in RF algorithm is the number of trees, and Oshio et al. stated that increasing the number of trees does not always mean the performance improvement [48]. Therefore, the number of trees is set as 10 and fewer trees indicates more generalizable of a trained model with regard to thousands of lesion cases in the DDSM database.

**Figure 1:**
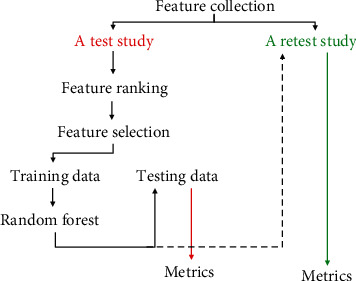
The proposed unified framework. It includes feature ranking, incremental feature selection, RF-based lesion classification, and performance evaluation, where features were precollected.

The unified framework is shown in Figure 1. It consists of feature ranking, incremental feature selection, RF optimization, and performance evaluation. Furthermore, feature ranking is based on the whole images in the study. In addition, after the RF-based model was built and evaluated on the testing samples, the model was further used to predict the malignance of the lesion images in the retest study. It is worth of note that parameters of FS methods are set as default.

### 2.6. Software Platform

Involved feature selection methods were implemented with MATLAB (MathWorks, Natick, MA, USA) where seven methods were from the Feature Selection Library [49], two methods (ROC and WILCOXON) were from the function *rankfeatures*, and one method (RELIEFF) was from the function *relieff*. Furthermore, the classifier RF was based on the function *randomForest* [50] in R (https://www.r-project.org/). The experiments were run on a personal laptop, and the laptop was equipped with dual Intel (R) Cores (TM) of 2.50 GHz and 8 GB DDR RAM. The implementation did not rely on any optimization or strategies for algorithm acceleration.

### 2.7. Statistical Analysis

Quantitative metrics were summarized as the mean ± standard deviation (SD) (MATLAB, MathWorks, Natick, MA, USA). Comparison between performance metrics is made with Wilcoxon rank-sum test or two sample *t*-tests when appropriate. All statistical tests are two sided, and *p* values less than 0.05 are defined as significant difference.

## 3. Results

### 3.1. Perceived Increase of AUC Values

**Figure 2:**
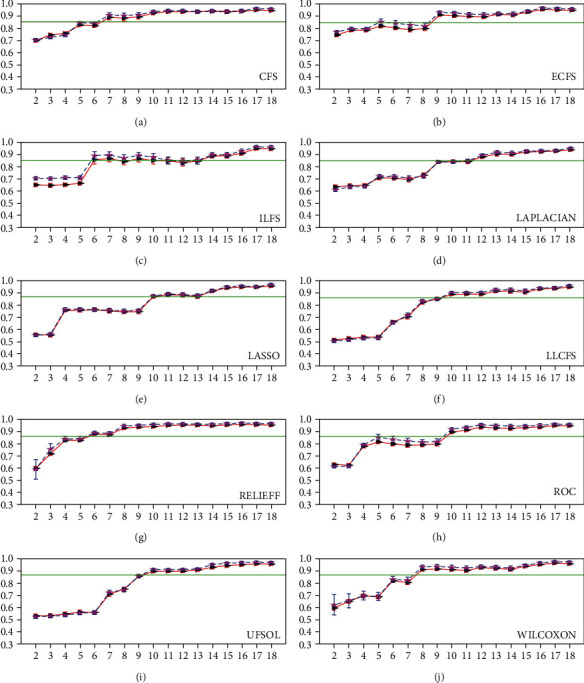
AUC. A baseline (green) of AUC equal to 0.85 is added to the plots. In each plot, the red solid line indicates the test result, while the blue dashed line shows the retest result. Besides, error bars are added. Please note that the figure can be enlarged to perceive details.

Figure 2 shows that the AUC values increased when features were added for mass lesion representation (red lines). When using top 2 features, both ECFS and CFS achieved AUC values that were averagely larger than 0.70 and AUC values from other FS methods that were larger than 0.60. Yet, the AUC values from UFSOL and LLCFS were <0.60, and the values did not show any obvious improvement until top 6 and 5 features were integrated in breast lesion classification, respectively. Compared to the baseline of AUC equal to 0.85 (green lines), both ILFS and RELIEFF obtained higher values when at least 6 features were used, followed by CFS (7 features) and WILCOXON (8 features), and other FS methods that required 9 to 10 features. In addition, for each diagnostic model, the error-bar plot of AUC in the retest study overlapped quite well with the plot in the test study.

### 3.2. Result Summary

Table 2 summarizes the number of features and corresponding performance metrics when a model achieves its AUC surpassing the baseline with the least feature number. It was observed that half of the methods required 10 or more features. In particular, when the first-time model exceeded the baseline, its SEN was higher than 0.85, while its ACC and SPE were relatively lower, indicating the potential false positive.

Table 3 summarizes the metric values when top two features are used for lesion representation. It was found that ECFS and CFS achieve AUC larger than 0.70, while three out of other eight methods reach AUC less than 0.60. We also found that ECFS, CFS, and ILFS reach SPE values larger than 0.50, while other methods tend to misclassify benign lesions into malignant ones.

The feature selection results are shown in Table 4 where the top-most important features of each model are highlighted in red. Frequency analysis of these features indicates that the 8^th^ feature and the 16^th^ feature are selected eight times, followed by the 4^th^ feature 7 times, while other features are equally used or less than 6 times.

## 4. Discussion

This study evaluated 10 FS methods in a unified framework for mammographic breast cancer diagnosis where RF is used as the classifier. Besides, the reliability of each diagnosis model was verified. Experimental results suggested that CFS has the ability to retrieve generally discriminative features. Based on the features ranked by CFS, the classification performance keeps improving. In addition, the CFS-based model achieved the 2^nd^ best performance when using top 2 features and it surpassed the baseline (AUC = 0.85) by using the top 7 features.

Some methods lead to unchanged or decreased performance at certain points when the number of features increases (Figure 2), which might be the selected features are redundant. These methods are ECFS, ILFS, LASSO, LLCFS, and ROC. In feature ranking, some methods omit the relationship between features. For instance, features *i_mean* and *i_median* (Appendix A) correlated well (Pearson's correlation coefficient, *p* = 0.99) and the two features are near each other in 8 out of 10 ranked feature lists (Table 4). Thus, it is helpful to remove the redundant features and continue to update diagnosis models in order to reach the optimal solution.

The use of a reasonable number of features is desirable in intelligent diagnosis since it implies a model lightweight computing; it is easy to interpret and can be generalized to other related applications. Investigation of top-ranked two features revealed that 7 out of 10 methods failed in distinguishing benign lesions from malignant ones (SPE < 0.5, Table 3). ECFS and CFS can achieve relatively good performance (AUC > 0.71, ACC > 0.63, SEN > 0.71, and SPE > 0.57). When the number of features increases, ILFS, RELIEFF, and CFS begin to exceed the baseline (Figure 2). On the other hand, except for AUC and SEN, other metrics have important roles since they allow for model evaluation from another perspectives. By comparing AUC, ACC, SEN, and SPE metrics, we found that most ACC and SPE values were lower than 0.80 when both AUC and SEN were larger than 0.85, which indicated that considerably benign lesions were misclassified and thereby, these patients would be exposed to unnecessary biopsies and would suffer from psychological anxiety.

Over recent years, FS has gained increasing attention. Notably, a series of models have been developed in radiomics [51–53]. Radiomics explores to represent one target from various perspectives where tens of thousands features can be crafted. Consequently, the selection of these discriminative features is a crucial, indispensable, but challenging step. On the other hand, the efficiency of feature subsets is hard to compare due to number of reasons such as FS being data dependent, which means that different data splitting may lead to change in the feature weights. Moreover, different FS methods might lead to distinct results because of theoretical frameworks, and this study obtained ten different selection results (Table 4).

This study has several limitations. First, few features were considered. It is known that massive features can be handcrafted based on mass intensity, shape, and texture in various transformed domains [30, 51–53], while it might make FS become challenging if hundreds of thousands features are involved, in particular for high dimension but small sample data analysis [54]. Second, this study evaluated a total of 10 FS methods among which 8 methods belong to the filter method group. Since filter methods are independent of classifiers, it avoids classifier selection and thus, computes efficiently. On the other hand, if more wrapper and embedded methods are compared, the conclusion that CFS having better performance would be more strongly supported. However, it is worth noting that this imbalance of FS methods does not affect the use of the proposed framework. Third, RF performs as the classifier, since it is important in classification tasks due to its interpretability [21]. From the technical perspective, other MLCs, such as ANN and SVM, are also feasible [12, 17, 20, 21, 24, 30]. It is also desirable to investigate the effects of RF parameters on the lesion diagnosis. However, it might lead to massive result reports and thus, only the number of trees is empirically determined and other parameters are set as default. Last but not the least, how to choose a proper FS method is a long-term problem in the field of computer-aided diagnosis. It should be admitted that feature extraction, FS methods, and MLCs are closely related to the ultimate goal of breast cancer diagnosis. Depending on specific purposes, such as diagnosis accuracy, model simplicity, interpretability, and generalization capacity, the selection of features, FS methods, and MLCs is different. Fortunately, the proposed framework can be expanded to incorporate more features as radiomics, more FS methods, and MLCs for classification or diagnosis tasks. Therefore, it is promising that systematic and comprehensive analysis on additional mammographic databases could deepen our understanding of breast cancer diagnosis from mammographic images.

## 5. Conclusions

This study evaluated ten feature selection methods for breast cancer diagnosis based on the digital database for screening mammography, where the random forest served as the machine learning classifier. Different methods led to distinct feature ranking results, and the correlation-based feature selection method was found to have superior performance in general. The way to find discriminative features out of thousands of features is challenging but indispensable for intelligent diagnosis and thus, extra efforts should be made towards accurate and efficient feature selection.

## Figures and Tables

**Table 1:** Feature selection methods.

ID	Acronym	Class	Learning strategy
A	CFS	Filter	Unsupervised
B	ECFS	Filter	Supervised
C	ILFS	Filter	Supervised
D	LAPLACIAN	Filter	Unsupervised
E	LASSO	Embedded	Supervised
F	LLCFS	Filter	Unsupervised
G	RELIEFF	Filter	Supervised
H	ROC	Filter	Unsupervised
I	UFSOL	Wrapper	Unsupervised
J	WILCOXON	Filter	Unsupervised

**Table 2:** Performance comparison. The metric values in bold come from the test study, while the values in the line below are from the retest study with corresponding features and model.

	No.	AUC	ACC	SEN	SPE
CFS	7	0.867 ± 0.023	0.733 ± 0.035	0.883 ± 0.018	0.793 ± 0.023
		0.896 ± 0.020	0.724 ± 0.035	0.900 ± 0.018	0.806 ± 0.022
ECFS	9	0.887 ± 0.018	0.739 ± 0.028	0.894 ± 0.011	0.806 ± 0.014
		0.926 ± 0.013	0.717 ± 0.034	0.915 ± 0.012	0.816 ± 0.017
ILFS	6	0.866 ± 0.028	0.678 ± 0.044	0.854 ± 0.030	0.763 ± 0.031
		0.907 ± 0.025	0.665 ± 0.043	0.884 ± 0.027	0.779 ± 0.029
LAPLACIAN	12	0.863 ± 0.018	0.730 ± 0.030	0.880 ± 0.013	0.790 ± 0.016
		0.891 ± 0.013	0.716 ± 0.028	0.893 ± 0.011	0.799 ± 0.014
LASSO	10	0.858 ± 0.020	0.685 ± 0.030	0.851 ± 0.013	0.763 ± 0.016
		0.862 ± 0.019	0.692 ± 0.025	0.856 ± 0.011	0.772 ± 0.013
LLCFS	10	0.855 ± 0.020	0.735 ± 0.027	0.876 ± 0.009	0.789 ± 0.013
		0.887 ± 0.014	0.714 ± 0.025	0.891 ± 0.009	0.796 ± 0.012
RELIEFF	6	0.855 ± 0.020	0.718 ± 0.026	0.868 ± 0.011	0.780 ± 0.013
		0.880 ± 0.015	0.695 ± 0.037	0.876 ± 0.012	0.782 ± 0.019
ROC	10	0.878 ± 0.019	0.728 ± 0.029	0.885 ± 0.013	0.796 ± 0.016
		0.919 ± 0.012	0.706 ± 0.035	0.908 ± 0.013	0.807 ± 0.018
UFSOL	10	0.858 ± 0.020	0.731 ± 0.028	0.877 ± 0.011	0.788 ± 0.013
		0.889 ± 0.016	0.709 ± 0.029	0.892 ± 0.009	0.794 ± 0.014
WILCOXON	8	0.887 ± 0.019	0.726 ± 0.027	0.890 ± 0.013	0.799 ± 0.015
		0.925 ± 0.013	0.707 ± 0.036	0.910 ± 0.013	0.810 ± 0.019

**Table 3:** Performance comparison when using top two features for lesion representation.

	No.	AUC	ACC	SEN	SPE
CFS	2	0.711 ± 0.012	0.636 ± 0.013	0.714 ± 0.027	0.572 ± 0.030
		0.715 ± 0.011	0.642 ± 0.012	0.718 ± 0.019	0.573 ± 0.026
ECFS	2	0.734 ± 0.013	0.660 ± 0.012	0.755 ± 0.026	0.581 ± 0.024
		0.759 ± 0.010	0.677 ± 0.011	0.785 ± 0.018	0.579 ± 0.021
ILFS	2	0.678 ± 0.012	0.606 ± 0.012	0.698 ± 0.023	0.530 ± 0.026
		0.724 ± 0.011	0.635 ± 0.011	0.752 ± 0.016	0.529 ± 0.025
LAPLACIAN	2	0.649 ± 0.014	0.603 ± 0.012	0.738 ± 0.025	0.492 ± 0.024
		0.626 ± 0.014	0.590 ± 0.011	0.737 ± 0.023	0.458 ± 0.020
LASSO	2	0.557 ± 0.014	0.526 ± 0.013	0.651 ± 0.025	0.422 ± 0.028
		0.552 ± 0.010	0.525 ± 0.010	0.653 ± 0.023	0.410 ± 0.023
LLCFS	2	0.517 ± 0.013	0.499 ± 0.013	0.645 ± 0.028	0.379 ± 0.024
		0.507 ± 0.012	0.498 ± 0.011	0.648 ± 0.025	0.363 ± 0.025
RELIEFF	2	0.611 ± 0.013	0.568 ± 0.014	0.689 ± 0.022	0.486 ± 0.028
		0.604 ± 0.073	0.574 ± 0.066	0.668 ± 0.021	0.490 ± 0.129
ROC	2	0.632 ± 0.013	0.582 ± 0.013	0.694 ± 0.025	0.491 ± 0.027
		0.616 ± 0.011	0.571 ± 0.011	0.716 ± 0.021	0.440 ± 0.034
UFSOL	2	0.543 ± 0.015	0.514 ± 0.012	0.654 ± 0.027	0.399 ± 0.021
		0.527 ± 0.013	0.513 ± 0.011	0.652 ± 0.024	0.388 ± 0.023
WILCOXON	**2**	0.605 ± 0.015	0.563 ± 0.015	0.686 ± 0.024	0.461 ± 0.028
		0.629 ± 0.075	0.587 ± 0.069	0.679 ± 0.020	0.505 ± 0.133

**Table 4:** Feature selection results. The top-most important features that achieve AUC larger than 0.85 are in bold to each FS method.

	The most to the least important features																	
CFS	**16**	**7**	**14**	**3**	**11**	**5**	**15**	6	2	8	13	17	10	9	1	4	12	18
ECFS	**8**	**9**	**17**	**4**	**10**	**2**	**1**	**16**	**12**	3	14	6	13	15	7	11	5	18
ILFS	**11**	**14**	**18**	**5**	**3**	**15**	13	1	4	2	10	6	9	7	16	12	8	17
LAPLACIAN	**8**	**5**	**4**	**3**	**9**	**2**	**1**	**16**	**7**	**18**	**6**	**11**	15	10	13	14	17	12
**LASSO**	**17**	**18**	**15**	**13**	**6**	**16**	**4**	**1**	**2**	**8**	9	5	3	7	11	14	10	12
LLCFS	**3**	**5**	**4**	**2**	**1**	**8**	**9**	**7**	**16**	**11**	18	6	15	10	14	13	17	12
RELIEFF	**10**	**14**	**11**	**7**	**18**	**8**	4	12	3	9	13	17	16	6	15	5	1	2
ROC	**9**	**17**	**4**	**8**	**10**	**2**	**1**	**16**	**3**	**12**	11	15	6	14	13	18	7	5
UFSOL	**9**	**1**	**2**	**3**	**5**	**4**	**8**	**16**	**7**	**11**	18	6	17	12	15	10	13	14
WILCOXON	**10**	**16**	**9**	**17**	**4**	**12**	**6**	**8**	14	2	1	13	3	18	7	11	15	5

## Data Availability

The data and toolboxes are available online. The data used to support the findings of this study are from http://www.eng.usf.edu/cvprg/Mammography/Database.html; the Feature Selection Library is https://www.mathworks.com/matlabcentral/fileexchange/56937-feature-selection-library; and the toolbox DDSM Utility from https://github.com/trane293/DDSMUtility is for data format transformation.

## Abbreviations

FS: Feature selection
AUC: The area under the curve
ACC: Accuracy
SEN: Sensitivity
SPE: Specificity
MLC: Machine learning classifier
SVM: Support vector machine
RF: Random forest
ANN: Artificial neural network
DDSM: Digital database for screening mammography
PGM: Portable gray map
LJPEG: Lossless joint photographic experts group
GLCM: Grey-level cooccurrence matrix
CFS: Correlation-based feature selection
ECFS: Feature selection via eigenvector centrality
EC: Eigenvector centrality
ILFS: Infinite latent feature selection
LAPLACIAN: Laplacian score
LASSO: Least absolute shrinkage and selection operator
LLCFS: Feature selection using local learning-based clustering
LLC: Local learning-based clustering
ROC: Receiver operating characteristic
UFSOL: Unsupervised feature selection with ordinal locality
WILCOXON: Wilcoxon rank-sum test
TP: True positive
FN: False negative
TN: True negative
FP: False positive
SD: Standard deviation.
